# Mucositis and salivary antioxidants in patients undergoing bone marrow transplantation (BMT)

**DOI:** 10.4317/medoral.19062

**Published:** 2014-03-08

**Authors:** Evelin Bachmeier, Marcelo A. Mazzeo, María M. López, Jorge A. Linares, Gustavo Jarchum, Fernando M. Wietz, Ana B. Finkelberg

**Affiliations:** 1 Dentist. Assistant Professor. Chair of Clinical Stomatology “A” and Chair of Physiology, Faculty of Odontology. Córdoba National University; 2 Doctor in Dentistry. Assistant Professor. Chair of Physiology. Córdoba National University; 3 Dentist. Assistant Professor. Chair of Physiology. Faculty of Odontology. Córdoba National University; 4 Biochemist. Chair of Physiology. Córdoba National University; 5 Medical Oncologist. Head of the Oncohematology Department, Sanatorio Allende, Córdoba. Odontologist. Assistant Professor. Chair of Physiology. Faculty of Odontology. Córdoba National University; 6 Dentist. Assistant Professor. Chair of Physiology. Faculty of Odontology. Córdoba National University; 7 Doctor in Biology. Full Professor. Chair of Physiology. Faculty of Odontology. Córdoba National University

## Abstract

Objectives: High doses of chemotherapy generate DNA damage in patients undergoing bone marrow transplantation (BMT), due to the production of reactive oxygen species (ROS). In order to evaluate the local defensive effectiveness of the patient undergoing BMT, the concentrations of the antioxidants superoxide dismutase (SOD) and uric acid (UA) were measured in saliva. Study Design: Basal saliva samples were collected from 20 patients undergoing BMT at the Oncology Department, Sanatorio Allende (Córdoba), in the stages: initial, prior to conditioning therapy (I); middle: 7 to 10 days after BMT (M) and final stage, 30 days after discharge from isolation (F). SOD levels were determined using a RANDOX kit (RANSOD superoxide dismutase manual), and for uric acid enzymatic UOD / PAP spectrophotometric method, ( Trinder Color Kit , Wiener Lab) was used. Results: 85% of the patients developed oral mucositis. SOD concentration in the M stage was significantly higher (*p*<0.01) compared with stage I, and it reversed in stage F. UA concentration was significantly lower (*p*<0.001) in stage M compared with stage I, and in stage F it recovered the initial values. Conclusions: SOD increase in stage M coincided with the appearance of mucositis, which could be interpreted as a defensive mechanism of saliva against oxidative stress produced by chemotherapy. UA decrease in stage M would favour the development of higher degrees of mucositis.

** Key words:**Bone marrow transplantation, mucositis, superoxide dismutase, uric acid.

## Introduction

Highly immunosuppressive and toxic treatments prior to the bone marrow stem cells transplantation (BMT) generate reactive oxygen species (ROS), which damage the DNA of the oral epithelium and submucosa.

This damage generates clinical and histological alterations in oral mucosa and periodontal tissues, as well as dysfunctions in the salivary glands and changes in saliva (1).

The current literature shows isolated and controversial results related to the side effects of different oncologic drugs on the oral cavity (2).

Likewise, the degree of incidence and severity of the complications is not clear, which makes difficult to consolidate palliative supportive treatments of the damage.

Most of the antineoplastic drugs act indiscriminately on the basal cells of oral epithelium, altering its renewal capacity. This, leads to the appearance of a series of systemic and local side effects such as mucositis, xerostomia, infections and hemorrhage.

Mucositis is an inflammatory reaction that may affect all the gastrointestinal mucosa, with high prevalence in the oropharyngeal area (3-6).

Mucositis is associated with a significant increase in the morbidity, producing a delay or even the interruption of the antineoplastic treatment as well as an increase in the therapeutic costs (7,8).

The etiopathogenesis of oral mucositis is not totally clear. However, series of risk factors associated with its appearance have been described, which are related mainly with the type of oncological treatment and with patient individual factors. In patients undergoing chemotherapy, the incidence and severity of toxic mucositis could be determined by the dose and the scheme of administration. Normally appears 3 to 5 days after therapy has been started, it reaches a maximum between 7 to 10 days, and resolves in the following 5 to 7 days, unless it is complicated by infection or hemorrhage (9,10).

Clinical signs of mucositis include epithelial atrophy, erosions and ulcerations of the oral mucosa, which increases the risk of systemic infection. Pain is often intense and could interfere with basic oral functions such as phonation, swallowing and eating (11), and in some cases it requires the administration of opioid analgesics.

Immunosuppression induced by chemotherapy during the conditioning period prior to bone marrow transplantation secondarily alters the capacity to respond to antigenic stimuli. The severity of the mucositis in these patients could be related with the degree of immunosuppression.

Recognizing the need to develop more effective and biological based therapies, recent research has focused on the pathobiology of mucositis. In its initial phase it involves direct damage to DNA and other cell components, generating ROS, which causes a cascade of biological events. In order to neutralize them, the organism has powerful antioxidant systems, both enzymatic and non-enzymatic (12).

The defensive battery provided by saliva is made of immunoenzymatic agents such as lysozyme, Ig A, salivary amylase and antioxidant substrates with a certain catalytic effect such as peroxidase, Superoxide Dismutase (SOD) and Uric Acid (UA) responsible for 70% of the antioxidant potential of saliva. The presence of each one of these antioxidants in saliva is conditioned mainly by glandular production, especially that of the parotid gland as in the case of SOD. The verification carried out by Kohen in 1992, that saliva modifies its enzymatic profile according to the humor reducing needs under the circumstances of oxidative stress, modified the then prevailing idea that it is a liquid of stable composition (13). Inflammatory responses provide a huge amount of free radicals, especially superoxide anion and H2O2. The salivary enzymatic production of antioxidants is eminently of glandular origin, being susceptible of increasing its concentration in different types of inflammatory disorders (14,15). The identification of antioxidants in tissues, blood and saliva provides an idea of the effective local or systemic defensive systems.

Based on these antecedents, the aim of our work was to evaluate the effective local defense of patients with mucositis undergoing BMT by measuring salivary antioxidants such as SOD and UA.

## Material and Methods

A longitudinal observational study was carried out, in a population of 20 patients admitted to the Department of Oncology, Sanatorio Allende, during 2009-2012, with prescription of BMT.

Exclusion criteria for this study were: patients under the age of 18 and over the age of 80, pregnant patients, patients with previous radiotherapy which had affected the craniofacial area, and tumors in the head and neck.

Informed consent was obtained from each participant, the study was approved by the Medical Ethics Committee of Sanatorio Allende and it was inscribed in REPIS (Provincial Register of Research on Human Health) under number 1189 (15/04/09).

-The following oral health indexes were registered.

Plaque index aimed at quantifying plaque deposits in the gingival margin (16).

Löe and Silness index: to evaluate the degree of gingival inflammation (17).

Simplified hemorrhage index: It indicates positive or negative bleeding, after probing the gingival sulcus with a periodontal probe (18).

Sulcus depth index (<4mm deep) or pocket (= > 4mm deep) of vestibular surface of the dental elements in both arches.

-Stomatological examination and saliva collection 

The stomatological examination and the collection of saliva samples were carried out at the following times.

A) Initial stage: prior to chemotherapy

B) Middle stage: during the isolation period, between the seventh and tenth day after BMT.

C) Final stage: Thirty days after finishing the isolation period.

Stomatological examination: an examination of the soft tissues of the oral cavity and annexed structures was made. The stomatological lesions were semiographed and an iconography of them was made.

Diagnostic and characterization of mucositis was performed following the WHO scale (19). 

-Collection of basal saliva.

Patients in a fasting state or at the first or second hour after breakfast, resting, sitting and silent, were asked to wash their mouth with distilled water. For 5 minutes, the saliva that formed and accumulated in the mouth was collected in a disposable centrifugal conic plastic tube previously weighted. Every patient prior to the BMT was considered as control group.

The material obtained was carried in a hermetically sealed container with freezing gel, at -4 C º. Then it was weighted, centrifuged, its pH was determined and it was conserved at - 18 Cº for its subsequent biochemical analysis (20).

-Sample analysis.

These were processed and analyzed at the laboratory of the Physiology Chair, Odontology Faculty, U.N.C.

In order to determine SOD, a RANDOX kit was used (RANSOD Superoxide Dismutases Manual), and for UA an enzymatic UOD / PAP spectrophotometric method (Trinder Color Kit , Wiener Lab) was used.

Statistical Analysis: data were analyzed by Student “T” test for paired data to compare the different stages of treatment, setting a *P* value < 0.05 for statistical significance.

## Results

65% Of the patients included in the sample were females. Patients were between 27 and 71 years- old, with a mean age of 44 years- old. Other characteristics of the patients included in the sample are shown in  Table 1.

**Table 1:**
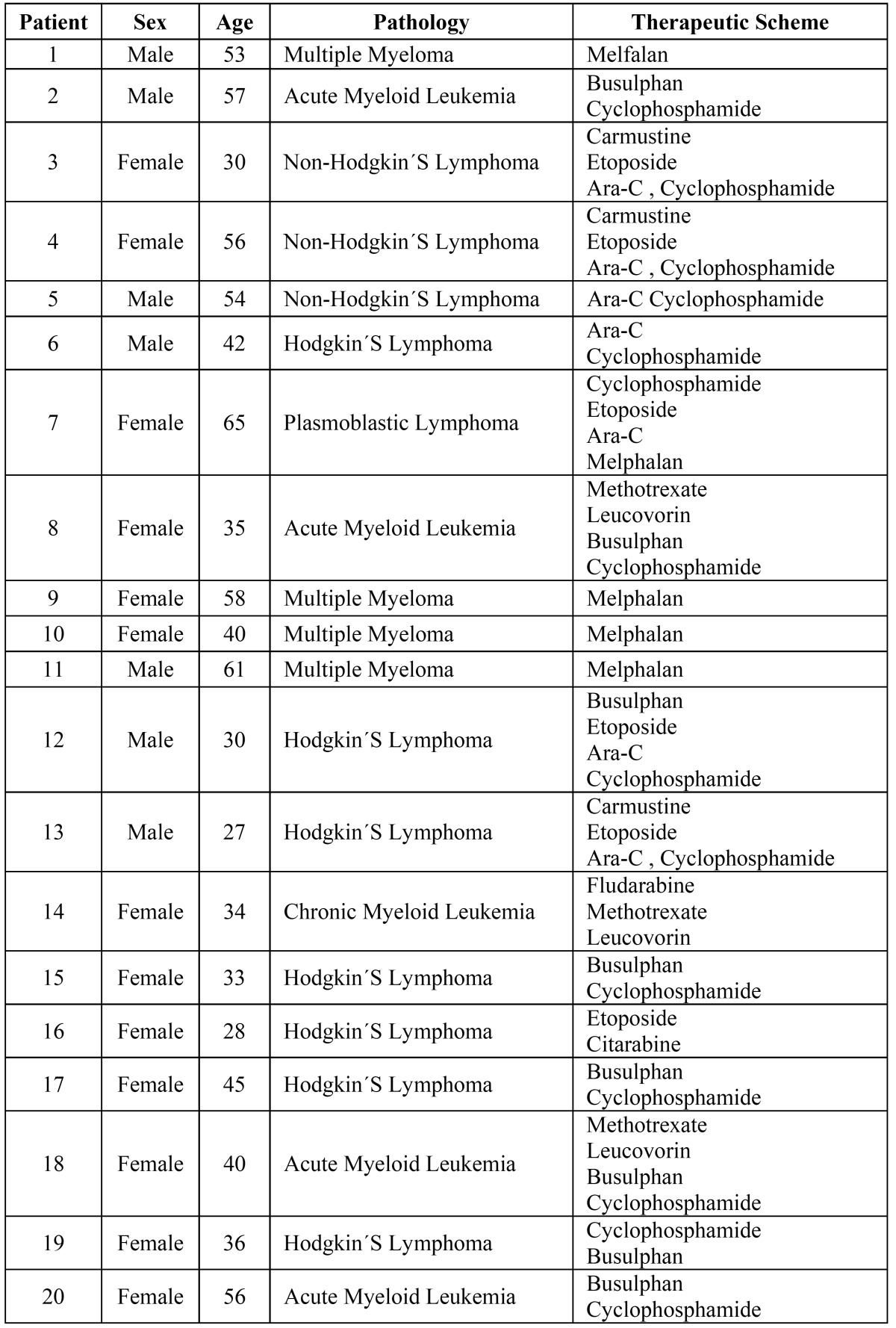
Characteristics of patients of the sample.

85% of the patients observed developed oral mucositis. In relation to the degree of severity, 50% showed mucositis grade 1, 15.78% grade 2 and 15,78% grade 3, while 5.26% showed grade 4.

**Figure 1:**
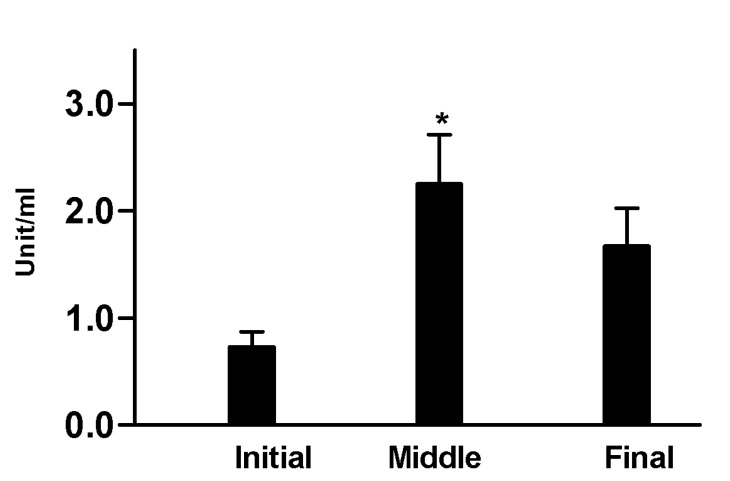
Concentration of SOD in the different stages of the study. Middle stage 2.25U/ml ± 1.30 vs. initial stage 0.73 U/ml±0.42 and final stage 1.67 U/ml ±0.94. (*) *p*<0,01.

The samples of basal saliva analyzed showed a significant increase of SOD during the middle stage, 2.25 U/ml ± 1.30 compared to initial 0.73 U/ml ± 0.42 and final 1.67 U/ml ± 0.94 (*p*<0.01) stages (Fig. 1).

**Figure 2:**
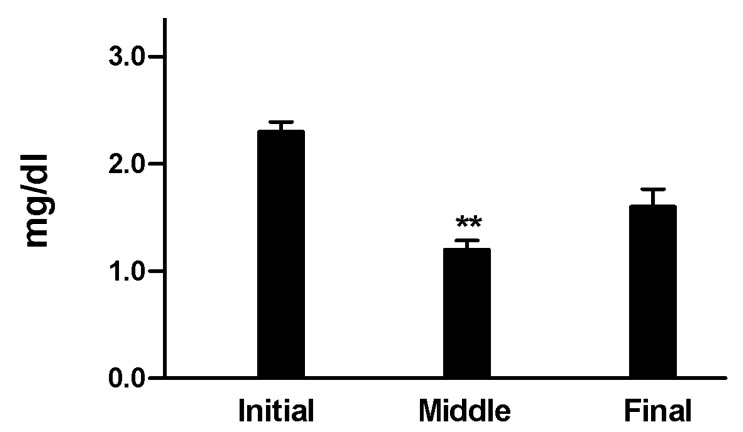
Concentration of UA in the different stages of the study. Middle stage 1,17mg/dl ±0,22 vs. initial stage 2,28 mg/dl±0,5 and final stage 1,58mg/dl ±0.33. (**) *p*<0.001.

UA showed a significant decrease in the middle stage 1.17 mg/dl ± 0.22 compared to the initial 2.28 mg/dl ± 0.5 and final 1.58 mg/dl ± 0.33 stages respectively (*p*<0.001) (Fig. 2).

Oral health indexes yielded the following results.

Gingival inflammatory response, evaluated by Löe and Silness rates, increased in the middle compared to the initial stage (*p*< 0.01), and reverted by the final stage. The simplified bleeding index did not exhibit statistically significant changes and plaque index revealed an increase in the middle stage compared with the initial stage.

Depth index of the vestibular sulcus of anterior and posterior elements significantly increased in the middle phase but did not revert at the end of the treatment (*p* <0.01).

## Discussion

The identification of antioxidants in tissues, blood and saliva and other body fluids provides an idea of the local or systemic defensive effectiveness of the individual (21).

In this sense, Nagler *et al*. state that saliva constitutes an important defensive line against oxidative stress (22).

Kim *et al*. classify the salivary antioxidants in three large groups, according to their function. The first group is formed by preventive antioxidants, which are those which inhibit the production of free radicals, such as SOD, carotenoids, catalase, glutathione peroxidase, transferrin, albumin and haptoglobin. Secondly, we find “sweeping” antioxidants, such as vitamin A and E, UA, albumin and bilirubin, which eliminate free radicals in order to inhibit the starting and spreading of cell damage. Finally, enzymes such as proteases, transferase, lipases, etc, repair the damage caused in the tissues (23-24).

Nagler *et al*. consider that the antioxidant salivary system is formed by an enzymatic component, where the main exponent is SOD and a non-enzymatic component, represented mainly by UA.

The production of SOD in saliva is of glandular origin, mainly parotideal. Yamamoto et al. showed that this enzyme is capable of increasing in response to different inflammatory reactions. In fact, some studies have demonstrated an increase in the concentration of SOD in processes such as tonsillitis, pulpitis, periodontitis and peri-implantitis (25-27).

There are few studies corcerning the relationship between SOD and inflammation in the oral cavity or periodontium, and the results are conflicting. Ellis *et al*. state that a significative reduction of SOD activity was found in gingival tissue adjacent to deep periodontal pockets (28,29). Other studies had indicated that total salivary antioxidants activity remains at the same level in periodontal disease, or is reduced (30,31). Canakci *et al*. had demonstrated that SOD activity both in saliva was lower in patients with periodontal disease compared to healthy subjects (32).

On the other hand, Wei *et al*. stated that SOD was significantly higher in the chronic periodontitis patients compared to the healthy patients of the control group (33).

Nagler studied the antioxidant profile of human saliva in healthy adults and determined that normal SOD values are 0.79 U/ml for basal saliva y 0.80 U/ml for the stimulated one. In our work on oncologic patients, we observed a significant increase in the identifications of SOD during the middle stage, coinciding with the beginning of clinical manifestations of oral mucositis. This increase in the concentration of SOD could be interpreted as a defense mechanism of saliva against oxidative stress produced by chemotherapy at high doses employed during the conditioning stage prior to bone marrow transplantation.

Considering the results of other groups as we discussed above, we consider that this increased activity of SOD, at least in part, also may be due to the changes in periodontal indexes during the middle stage. However, reports on the relationship between the antioxidant status and periodontal diseases have been controversial. We consider that further investigation is needed in order to clarify possible correlation between salivary SOD levels, grades of mucositis and periodontal status.

On the other hand, Halliwell and Moore reported that the most important antioxidant of saliva is UA, responsible for 70% of the antioxidant activity, followed by albumin and ascorbic acid (27,31).

Goll and Mookerjee observed that the concentrations of this antioxidant in total saliva correlate significantly with those of plasma (34).

Nagler reported that the normal values of UA were: 2.87 mg/dl in basal saliva and 10.5 mg/dl for stimulated saliva. In our study we observed a significant decrease of UA during the middle stage, which could favor the appearance and progression of oral mucositis.

According to Bibi *et al*., an increase in the production of salivary SOD could be considered a partial compensatory response when there is reduction of the concentrations of UA and oxidative stress in the oral cavity (35). As regards this, our results coincide with the above mentioned since they show an increase in the concentration of SOD accompanied by a decrease of the concentrations of UA during the middle stage (36-37).

Impaired oxidant/antioxidant balance is responsible for the tissue damage in BMT patients. Measuring some parameters such as salival SOD and UA, would allow to improve noninvasive methods in order to evaluate the antioxidant capacity in patients undergoing BMT. We propose that future studies on this issue should be done. The results of the present work may be useful in evaluating the antioxidant profile during conditioning chemotherapy and, in case of being necessary, founding new therapies using antioxidant supplementation or nutritional prescriptions that might elevate antioxidant local and systemic defences in BMT patients.

## Conclusions

The determination of the salivary concentrations of antioxidants such as SOD and UA provides a non-invasive method which allows evaluating the defensive capacity of the oral mucosa in oncologic patients under treatment with chemotherapy drugs at high doses.

At this study, the increase of the levels of SOD in patients that developed mucositis could be interpreted as a defense mechanism in the presence of the injury that chemotherapy represents to the cells of the oral mucosa, while a decrease of UA would allow the starting, progression and the tissue damage.

By virtue of the results obtained, we consider that it is necessary to do future research which permit to obtain further knowledge about these and other antioxidant substances in saliva and its correlation with oral mucositis.

The integration of dentists in the oncology team becomes important in the study, prevention, diagnosis and treatment of the oral complications, in order to improve the quality of life in patients undergoing chemotherapy.
